# Methemoglobin and nitric oxide therapy in Ugandan children hospitalized for febrile illness: results from a prospective cohort study and randomized double-blind placebo-controlled trial

**DOI:** 10.1186/s12887-016-0719-2

**Published:** 2016-11-04

**Authors:** Andrea L. Conroy, Michael Hawkes, Kyla Hayford, Laura Hermann, Chloe R. McDonald, Suparna Sharma, Sophie Namasopo, Robert O. Opoka, Chandy C. John, W. Conrad Liles, Christopher Miller, Kevin C. Kain

**Affiliations:** 1 Depatment of Medicine, University of Toronto, Toronto, Canada; 2 Sandra A. Rotman Laboratories, Sandra Rotman Centre for Global Health, University Health Network-Toronto General Hospital, University of Toronto, Toronto, Canada; 3 Division of Pediatric Infectious Diseases, University of Alberta, Edmonton, Canada; 4 Institute of Medical Sciences, University of Toronto, Toronto, Canada; 5 Department of Pediatrics, Jinja Regional Referral Hospital, Jinja, Uganda; 6 Department of Paediatrics and Child Health, Mulago Hospital, Makerere University, Kampala, Uganda; 7 Department of Pediatrics, Indiana University School of Medicine, Indianapolis, IN USA; 8 Department of Medicine, University of Washington, Seattle, WA 98195 USA; 9 Department of Respiratory Medicine, Faculty of Medicine, University of British Columbia, Vancouver, Canada; 10 Tropical Disease Unit, Division of Infectious Diseases, Department of Medicine, University of Toronto, Toronto, Canada; 11 MaRS Centre, TMDT, 10th floor 10-351, Toronto, ON M5G1L7 Canada

**Keywords:** Pediatrics, Methemoglobin, Inhaled nitric oxide, Malaria, Anemia, Metabolic acidosis, Oxygen delivery, Fever, Uganda

## Abstract

**Background:**

Exposure of red blood cells to oxidants increases production of methemoglobin (MHb) resulting in impaired oxygen delivery to tissues. There are no reliable estimates of methemoglobinemia in low resource clinical settings. Our objectives were to: i) evaluate risk factors for methemoglobinemia in Ugandan children hospitalized with fever (study 1); and ii) investigate MHb responses in critically ill Ugandan children with severe malaria treated with inhaled nitric oxide (iNO), an oxidant that induces MHb in a dose-dependent manner (study 2).

**Methods:**

Two prospective studies were conducted at Jinja Regional Referral Hospital in Uganda between 2011 and 2013. Study 1, a prospective cohort study of children admitted to hospital with fever (fever cohort, *n* = 2089 children 2 months to 5 years). Study 2, a randomized double-blind placebo-controlled parallel arm trial of room air placebo vs. 80 ppm iNO as an adjunctive therapy for children with severe malaria (RCT, *n* = 180 children 1–10 years receiving intravenous artesunate and 72 h of study gas). The primary outcomes were: i) masimo pulse co-oximetry elevated MHb levels at admission (>2 %, fever cohort); ii) four hourly MHb levels in the RCT.

**Results:**

In the fever cohort, 34 % of children admitted with fever had elevated MHb at admission. Children with a history of vomiting, delayed capillary refill, elevated lactate, severe anemia, malaria, or hemoglobinopathies had increased odds of methemoglobinemia (p < 0.05 in a multivariate model). MHb levels at admission were higher in children who died (*n* = 89) compared to those who survived (*n* = 1964), *p* = 0.008. Among children enrolled in the iNO RCT, MHb levels typically plateaued within 12–24 h of starting study gas. MHb levels were higher in children receiving iNO compared to placebo, and MHb > 10 % occurred in 5.7 % of children receiving iNO. There were no differences in rates of study gas discontinuation between trial arms.

**Conclusions:**

Hospitalized children with evidence of impaired oxygen delivery, metabolic acidosis, anemia, or malaria were at risk of methemoglobinemia. However, we demonstrated high-dose iNO could be safely administered to critically ill children with severe malaria with appropriate MHb monitoring.

**Trial registration:**

ClinicalTrials.gov Identifier: NCT01255215 (Date registered: December 5, 2010).

## Background

An estimated 200 million malaria infections occur every year, resulting in an estimated 1.2 million deaths [1], the majority of which are attributable to *Plasmodium falciparum* infection. Despite the availability of effective artemisinin-based antimalarial therapies, mortality rates remain high in severe malaria (8–20 % in children), suggesting that therapies targeting the parasite alone are insufficient in individuals with established manifestations of severe disease. One potential strategy to reduce mortality rates is to identify adjunctive therapies that target deleterious host immune responses (reviewed in [2, 3]). The endothelium, acting as a ‘biosensor’, is increasingly being recognized as a critical regulator of vascular integrity in life-threatening infections characterized by systemic inflammation, like sepsis and severe malaria, and therefore represents a promising target for adjunctive therapy [4].

One strategy to promote endothelial integrity is to increase bioavailable nitric oxide (NO), a gaseous free radical produced by the conversion of L-arginine to L-citrulline through a family of nitric oxide synthase enzymes [5]. A number of human studies have reported associations between reduced bioavailable NO and malaria disease severity [6–8]. Preclinical data from an experimental model of cerebral malaria (CM) reported improved survival, reduced systemic inflammation and endothelial activation, and retained blood-brain-barrier integrity following administration of iNO [9, 10]. iNO is approved for human use (5–80 ppm), and is routinely used in North America and Europe for the treatment of persistent pulmonary hypertension or infant respiratory distress syndrome in term or near-term neonates [11, 12]. Based on these data, we sought to evaluate whether iNO would improve clinical recovery when administered in adjunct to standard anti-malarial therapy in a cohort of children with severe malaria [13, 14].

As iNO is systemically absorbed it combines with hemoglobin to form nitrosylhemoglobin, which is then oxidized to form methemoglobin (MHb) [15]. MHb is formed by oxidation of ferrous iron (Fe^++^) to ferric iron (Fe^+++^) within the heme moiety of hemoglobin (Hb), resulting in a functional impairment in the ability of Hb to transport oxygen and carbon dioxide [16]. As red blood cells (RBCs) are continuously bathed in oxygen, there is constant oxidation of Hb to MHb, but levels of MHb typically remain <1 % due to endogenous reduction systems [17]. Elevated levels of MHb (>10 %) can lead to clinical signs of hypoxemia ranging from mild headache to respiratory distress, cyanosis and death with increasing levels of MHb. Although methemoglobinemia typically occurs following ingestion or skin exposure to an oxidizing agent, it can also occur as a result of genetic, dietary or other factors [18].

In this study, we prospectively evaluated MHb levels in Ugandan children hospitalized with fever. Our objectives were three-fold: i) to determine MHb levels at admission among children hospitalized for febrile illness in Ugandan children; ii) to explore clinical and demographic factors associated with elevated MHb; and iii) to evaluate the safety and tolerability of high-dose inhaled nitric oxide in children with severe malaria using MHb. We assessed the first two objectives in a prospective observational study of 2089 febrile children admitted to hospital in a resource-constrained hospital in Eastern Uganda. The impact of iNO on MHb was assessed in a randomized double-blind placebo-controlled trial evaluating iNO as an adjunctive therapy for children with severe malaria where iNO was administered at 80 ppm continuously for up to 72 h. Although iNO is routinely administered in neonates at doses of 5–20 ppm, there are limited data on the effect of iNO on MHb levels at higher doses, and no data from pediatric populations in Africa.

## Methods

### Study site

Studies took place at the Jinja Regional Referral Hospital between July 2011 and August 2013 in Jinja, Uganda. The hospital serves a catchment area of 3 million people encompassing 12 districts in mid-eastern Uganda. The children’s unit has 100 beds and an average admission rate of 650 children per month. Malaria transmission in the Jinja area is moderate with an estimated entomological inoculation rate of six infective bites per person per year [19, 20]. Malaria is the most common admission diagnosis in the children’s unit.

### Study 1 design: prospective in-patient study of children hospitalized with non-malarial and malarial fever

Children aged 2 months to 5 years were eligible for the study if they had a documented fever or history of fever within the previous 48 h and were admitted to hospital by the attending physician. Children with diarrheal illness without any other symptoms of systemic infection were excluded from the study. At admission, information was collected on patient demographics, history of illness, and treatments. Daily follow-up was conducted by study personnel to determine clinical outcome. Methemoglobin was assessed using a Masimo SET® Rad-57™ pulse co-oximeter (Masimo Corporation, Irvine, CA), by experienced pediatric nurses and medical officers according to standard operating procedures. Malaria infection (lab-confirmed malaria) was defined using microscopy (Field’s stained thick blood smear examined by an experienced technician at the Jinja Hospital Laboratory using a light microscope) and/or rapid diagnostic tests (HRP2/pLDH positive or pLDH positive test, First Response MalariaAg. pLDH/HRP2 Combo Rapid Diagnostic Test, Premier Medical Corporation Limited, India) [21].

### Study 2 design: randomized double-blind placebo-controlled clinical trial comparing air versus high-dose iNO as an adjunctive therapy for severe malaria

Children aged 1 to 10 years with suspected severe malaria were screened in the emergency department at Jinja Regional Referral Hospital for inclusion in the trial. The trial is registered (ClinicalTrials.gov Identifier: NCT01255215). Children were eligible for the study if they had a positive malaria rapid diagnostic test in the presence of features of severe malaria [13]. After obtaining informed consent, children were randomized to receive either room air or iNO starting at 80 ppm by non-rebreather HiOx® face mask (CareFusion, CA) for 72 h (or until the child recovered and no longer tolerated the mask). Children were randomized using simple randomization using a computer generated list created by the unblinded team leader (ALC). Group assignment was recorded on a piece of paper and kept in sequentially sealed opaque envelops in a locked cabinet accessible only to un-blinded investigators. Following enrollment, malaria was confirmed using thick and thin Giemsa-stained peripheral blood smears assessed by light microscopy at the Makerere University-John’s Hopkins University (MU-JHU) Core Lab, which is a College of American Pathologists certified, quality-controlled central research laboratory in Kampala. All children received parenteral artesunate for severe malaria as described [22]. Children were excluded from the study if they had known chronic illnesses (e.g. renal, cardiac or hepatic diseases, epilepsy, cerebral palsy, clinical AIDS), hemoglobinopathies, severe malnutrition (<−3SD weight-for-age), severe malarial anemia (Hb <50 g/L) without any other signs of severe malaria, and baseline methemoglobinemia (>2 %) that did not resolve following patient stabilization.

Study gas (continuous iNO or room air placebo) was administered by an un-blinded research team not involved in patient care including: a trial manager to randomize children and start treatment gas, and a team of un-blinded study nurses to monitor gas delivery and potential toxicities. Study gas was temporarily discontinued if one of the following occurred: MHb >10 %; elevated inspired NO_2_ concentration >5 %; persistent hypoxemia; evolving respiratory distress; unexplained tachycardia; unexplained hypotension; any study drug related adverse event that, in the opinion of the investigator, made it unsafe for the subject to continue. Following temporary discontinuation of the study gas, there was the possibility of re-challenge following resolution of the adverse event. Treatment was permanently discontinued if there was refractory methemoglobinemia (MHb above 10 % despite re-starting iNO at a lower concentration following temporary discontinuation); hemoptysis; acute kidney injury; any study drug related adverse event that, in the opinion of the investigator, made it unsafe for the subject to continue; any study drug related adverse event requiring temporary discontinuation that recurred on re-challenge at the same or lower dose of iNO; or at the discretion of the subject or guardian; at the discretion of the investigator.

Baseline MHb levels were assessed at time of patient screening and were repeated following randomization. After the study gas was initiated, MHb levels were assessed on a four-hourly basis. Nurses kept detailed clinical record of any time off gas to accurately assess the time children were exposed to iNO.

### Statistical analysis

Data were analyzed using IBM SPSS 20, Stata 13 (College Station, TX) and GraphPad Prism 6. Demographic, clinical and laboratory characteristics of participants at enrolment were described using proportions for binary variables and mean or median values for continuous variables, as appropriate. Age and sex-standardized z-scores for height-for-age, weight-for-age and height-for-weight were calculated using the World Health Organization Anthro program (version 3.2.2, January 2011).

Baseline MHb levels were analyzed as a percentage or categorized as methemoglobinemia (>2 % vs. ≤2 %). Risk factors for methemoglobinemia at admission were evaluated using bivariate and multivariate logistic regression models. Model selection for the multivariate model was based on variables selected a priori (age) and all variables that predicted methemoglobinemia at an alpha level of ≤0.2 in bivariate logistic regression models. Final variable selection for the multivariate model balanced parsimony with model fit based on Hosmer-Lemeshow’s goodness of fit test, minimizing Akaike’s Information Criteria and Bayesian Information Criteria. Unadjusted and adjusted odds ratios are presented with 95 % confidence intervals.

## Results

Data were analyzed for 2089 children with known outcomes in the pediatric fever cohort (study 1) and 180 children in the iNO RCT (study 2). Median age was 1 year [IQR: 0, 2] in the fever cohort and 2 years [1, 3] in the iNO RCT (Table 1). Clinical characteristics at enrollment such as temperature, heart rate, oxygen saturation, capillary refill time and blood pressure were comparable in both cohorts. In the fever cohort (study 1), the median Blantyre coma score (BCS) was 5 [IQR: 5,5], 33 % presented with vomiting and 30 % presented with diarrhea. In the iNO RCT, the median BCS was 2 [2, 3] with 4 and 21 % presenting with vomiting and diarrhea, respectively. Sixty-seven percent of children in the pediatric fever cohort had lab-confirmed malaria (by thick film blood smear or positive RDT pLDH/HRP2 or pLDH alone) compared to 100 % in the iNO RCT, an enrollment criteria for the RCT. Twenty percent and 61 % of fever and iNO RCT cohorts, respectively, had severe anemia (Table 1). Median MHb levels at admission were 1.5 % [IQR: 0.7, 2.6] in the fever cohort and 1.7 % [1.2, 2.1] in the iNO RCT cohort (*p* >0.05). Sixty-two out of 547 (11.3 %) subjects were excluded from the iNO RCT due to elevated MHb at admission.

**Table 1:** Characteristics of Study Cohorts

	Fever Cohort (*n* = 2089)	iNO Trial Cohort (*n* = 180)
Age, years^a^	1 [0, 2]	2 [1, 3]
Male, % (#)	55 % (1134)	57 % (102)
Temperature, °C	37.9 (1.2)	37.9 (1.2)
Heart rate, bpm	159.2 (25.2)	160.7 (25.0)
Systolic BP	105.0 (15.9)	110.5 (20.2)
Diastolic BP	57.4 (13.4)	58.7 (13.6)
Respiratory rate	44 [36, 56]	48 [38, 62]
Vomiting	33 % (686)	4 % (8)
Diarrhea	30 % (614)	21 % (38)
Blantyre coma score	5 [5, 5]	2 [2, 3]
SpO2	98 [96, 100]	99 [98, 100]
Lactate (mmol/L)^b^	2.7 [1.9, 4.9]	3.6 [2.1, 6.4]
Lactate >5 mmol/L^b^	24 % (485)	24 % (43)
Capillary refill time		
<2 s	85 % (1724)	82 % (148)
2–3 s	10 % (194)	12 % (22)
>3 s	5 % (106)	6 % (10)
Lab-confirmed malaria^c^	67 % (1240)	100 %
Severe anemia^d^	20 % (428)	61 % (109)
Pretreatment with antibiotic	33 % (682)	43 % (74)
Pretreatment with antimalarial	46.2 % (956)	59 % (105)

^a^Mean (SD) for normally distributed variables. Median [IQR] for non-normally distributed variables. Number (%) for categorical variables

^b^Lactate was assessed using LactateScout in the fever cohort and i-STAT in the iNO trial as previously described [52]

^c^Positive by microscopy or RDT (HRP2/pLDH or pLDH)

^d^Severe anemia defined as hemoglobin less than 5 g/dL (hospital laboratory) or pallor by clinical assessment in the fever cohort and Hb <5 g/dL (reference laboratory) in the iNO trial cohort

### Study 1 cohort: risk factors of elevated MHb in a pediatric fever cohort

Among 2089 children admitted to the hospital with a fever (Table 2), 34 % had methemoglobinemia (MHb >2 %), 6 % had MHb levels above 7, and 3 % had MHb levels above 10 %. In bivariate analysis, multiple factors differed between children with vs. without methemoglobinemia (Table 2). In a multivariate logistic regression model, children with methemoglobinemia at presentation were significantly more likely to have vomiting (adjusted odds ratio (aOR) 1.36, 95 % CI: 1.09, 1.70), prolonged capillary refill time (aOR 1.36, 95 % CI: 1.11, 1.66) and elevated lactate levels (aOR 1.08, 95 % CI: 1.05, 1.11) after controlling for relevant demographic, clinical and laboratory results. The odds ratio of methemoglobinemia was two times higher in children with hemoglobinopathies (sickle cell anemia or glucose-6-phosphate dehydrogenase deficiency (G6PD)) (aOR 1.97, 95 % CI: 1.17, 3.32), or in severe anemia (aOR 1.99, 95 % CI: 1.51, 2.61). Children with lab-confirmed malaria (aOR 1.34, 95 % CI: 1.07, 1.69) also had an elevated risk of methemoglobinemia. Of children with lab-confirmed malaria, 54.6 % met WHO criteria for severe malaria, including: prostration, deep breathing, jaundice, hyperlactatemia, hypoglycemia, severe anemia, altered consciousness or hemoglobinuria. MHb levels were higher in children with severe malaria (*n* = 754; median 1.9 %, IQR, 0.8–4.1) compared to uncomplicated malaria (*n* = 627; median 1.4 %, IQR: 0.7–2.0), *p* <0.0001 by Mann-Whitney U test.

**Table 2:** Factors associated with methemoglobinemia in a pediatric fever cohort

	MHb ≤2 % *N* = 1364 (66 %)	MHb >2 % *N* = 689 (34 %)	Bivariate OR (95 % CI)	*P*-value	Multivariate OR (95 % CI)	*P*-value
Demographic characteristics						
Age, months	17 [9, 26]	18 [9, 30]	1.00 (1.00, 1.01)	0.268	1.00 (1.00, 1.01)	0.370
Age <6 months	98 (7.2)	53 (7.7)	1.08 (0.76, 1.52)	0.677		
Male (%)	734 (54.4)	382 (56.1)	1.07 (0.89, 1.29)	0.461		
Clinical findings at admission						
Fever (≥38^o^ C)	402 (39.1)	276 (27.6)	**0.59 (0.49, 0.72)**	**<0.001**	**0.74 (0.60, 0.92)**	**0.005**
Underweight, <-2 WAZ	303 (22.6)	150 (22.3)	0.97 (0.77, 1.21)	0.775		
Systolic BP	105.5 (15.3)	104.2 (7.0)	0.99 (0.99, 1.00)	0.094		
Diastolic BP	58.4 (13.3)	55.6 (13.4)	**0.98 (0.98, 0.99)**	**<0.001**	0.99 (0.98, 1.00)	0.130
Age-specific elevated respiratory rate, per min	693 (52.7)	398 (59.8)	**1.34 (1.11, 1.62)**	**0.002**		
Deep breathing	282 (20.7)	213 (31.0)	**1.72 (1.40, 2.12)**	**<0.001**		
Vomiting	399 (29.3)	272 (39.6)	**1.58 (1.30, 1.91)**	**<0.001**	**1.36 (1.09, 1.70)**	**0.007**
Diarrhea	424 (31.2)	176 (25.6)	**0.82 (0.64, 0.)**	**0.008**	0.82 (0.64, 1.04)	0.107
Blantyre coma score						
0 1 2 3 4 5	21 (1.6) 13 (1.0) 22 (1.6) 45 (3.4) 63 (4.7) 1173 (87.7)	26 (3.9) 9 (1.3) 25 (3.7) 41 (6.1) 48 (8.6) 514 (76.4)	**0.78 (0.71, 0.85)**	**<0.001**		
Capillary refill time						
< 2 seconds	1180 (89.5)	515 (77.0)				
2- <3 sec	90 (6.8)	102 (15.2)				
≥ 3 sec	49 (3.7)	49 (3.7)	**1.81 (1.52, 2.16)**	**<0.001**	**1.36 (1.11, 1.66)**	**0.003**
Pretreatment with antibiotics	428 (31.6)	240 (35.5)	1.19 (0.98, 1.45)	0.079		
Pretreatment with sulfadoxine pyremethamine	16 (1.2)	14 (2.1)	1.76 (0.85, 3.63)	0.125		
Subcostal retractions	236 (17.3)	172 (25.0)	**1.59 (1.27, 1.98)**	**<0.001**		
Laboratory test results at admission						
Lactate, mmol/L	2.5 [1.8, 4.0]	3.4 [2.2, 8.5]	**1.14 (1.11, 1.16)**	**<0.001**	**1.08 (1.05, 1.11)**	**<0.001**
Glucose, mmol/L	7.1 (2.3)	7.9 (3.3)	**1.11 (1.07, 1.15)**	**<0.001**		
Oxygen saturation (Sp0_2_)	98 [96, 100]	98 [95, 99]	**0.97 (0.95, 0.99)**	**0.015**		
Severe anemia, Hb < 5 g/dL or pallor	182 (13.3)	233 (33.8)	**3.32 (2.66, 4.14)**	**<0.001**	**1.99 (1.51, 2.61)**	**<0.001**
Suspected hemoglobinopathy	41 (3.0)	41 (6.0)	**2.04 (1.31, 3.18)**	**0.002**	**1.97 (1.17, 3.32)**	**0.011**
Lab-confirmed malaria^1^	883 (64.8)	498 (72.5)	**1.43 (1.17, 1.74)**	**0.001**	**1.34 (1.07, 1.69)**	**0.011**

Mean (SD) for normally distributed variables. Median [IQR] for non-normally distributed variables. Number (%) for categorical variables

^1^Positive by microscopy or RDT (HRP2/pLDH or pLDH)

Factors significantly associated with methemoglobinemia in bivariate or multivariate analysis in bold

Admission MHb levels were higher in non-survivors (*n* = 89; median 1.7 %, IQR: 0.8–4.6) compared to survivors (*n* = 1964; median 1.5 %, IQR: 0.7–2.5), *p* = 0.008 (Mann-Whitney U Test). Analysis of missing data showed that MHb values were more likely to be missing in non-survivors compared to survivors at 10.1 and 1.3 % respectively (reflecting the difficulty in getting pulse co-oximetry measurements in critically ill children with poor perfusion).

### Study 2 cohort: MHb levels in children receiving iNO as an adjunctive therapy for severe malaria

#### Overview of trial

Among children enrolled in the iNO RCT, 92 were randomized to receive placebo (room air) and 88 to receive iNO between July 2011 and June 2013. The mean time on gas was comparable between groups (mean (SD): placebo, 63.6 (19.9) hours; iNO, 61.9 (21.7) hours, *p* = 0.582). Gas was withdrawn for 31 children (12 children in the placebo arm and 19 in the iNO arm, *p* = 0.13). 10 children had gas temporarily discontinued for the following reasons: methemoglobin >10 %, *n* = 5; unexplained tachycardia, *n* = 1; investigator discretion, *n* = 4 (stridor, *n* = 2; resuscitation, *n* = 1; transfer to another hospital for transfusion, *n* = 1). 21 children had gas permanently discontinued for the following reasons: acute kidney injury, *n* = 10; guardian withdrew consent, *n* = 2; technical/power issues, *n* = 2; mask required for oxygen delivery, *n* = 1; recovery/refusal to tolerate the mask, *n* = 6. There were no differences between rates of temporary (placebo, *n* = 3 vs. iNO, *n* = 7; *p* = 0.21) or permanent discontinuation (placebo, *n* = 10 vs. iNO, *n* = 12; *p* = 0.57) of study gas between trial arms. However, MHb >10 % requiring temporary withdrawal of study gas only occurred in children receiving iNO (*n* = 5, 5.7 %), *p* = 0.026. Apart from elevated MHb, there were no other study drug-related adverse events listed in the product monograph (hypotension, atelectasis, hematuria, hyperglycemia, sepsis, infection, stridor, cellulitis) [23].

#### MHb response to iNO administration

**Fig. 1:**
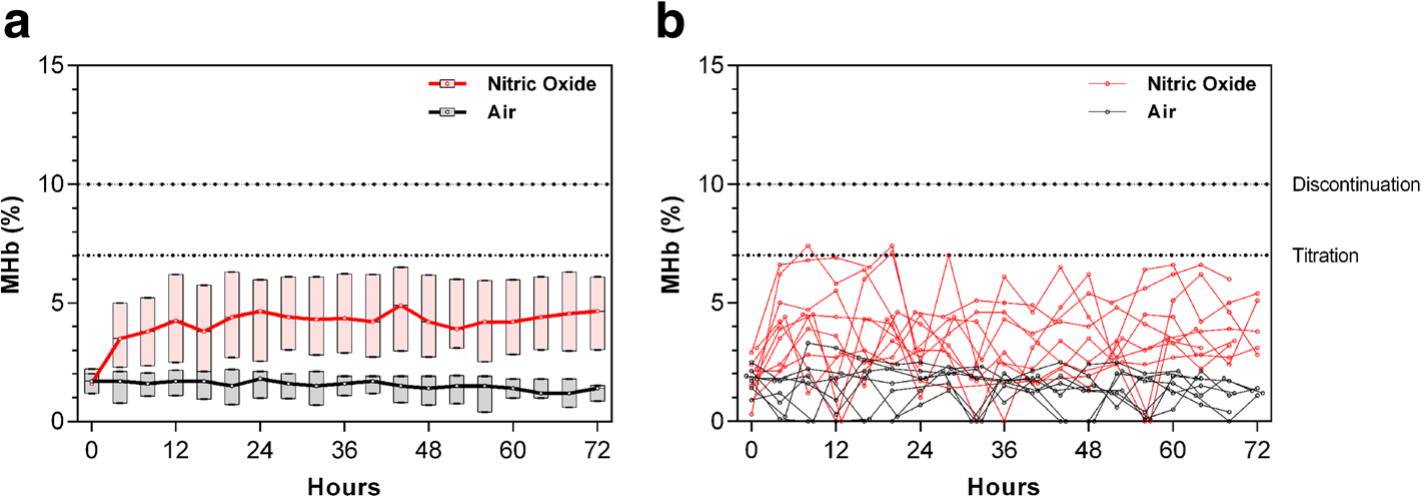
MHb levels in children with severe malaria randomized to room air or nitric oxide as an adjunctive therapy to intravenous artesunate. **a** Box and whisker plots showing the median (IQR) and 95 % CI for the trial arms at scheduled four hourly MHb checks. **b** Representative MHb plots for a random subset (10 %) of study participants (*n* = 7 placebo arm, *n* = 10 nitric oxide arm)

**Fig. 2:**
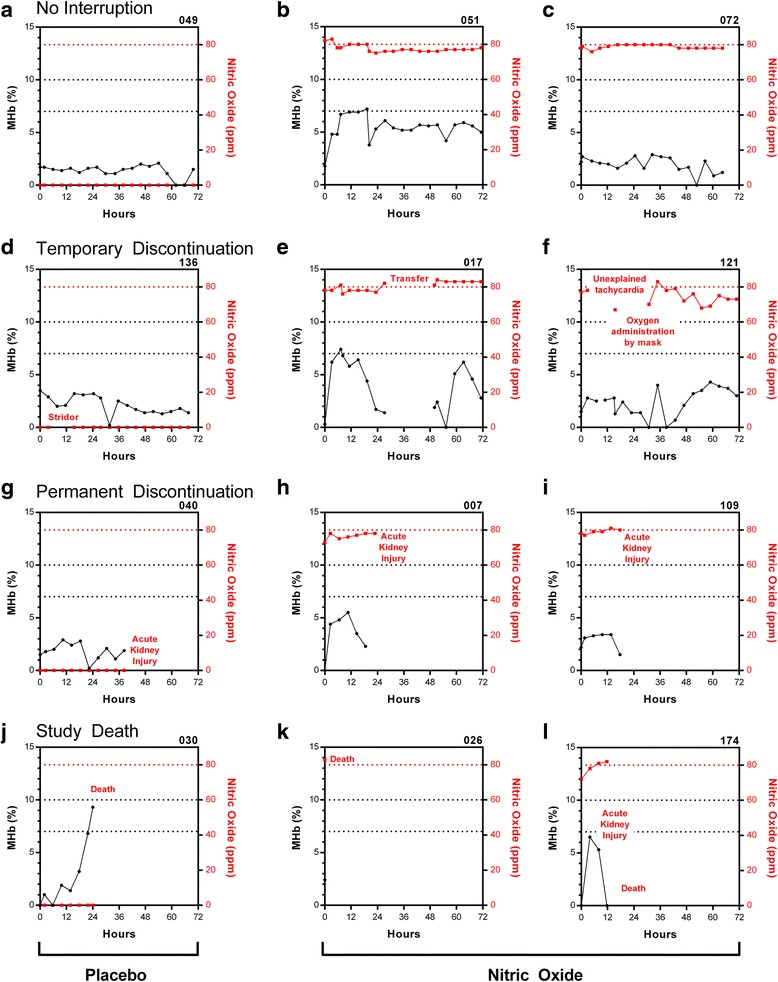
Representative graphs of methemoglobin kinetics and nitric oxide concentrations administered to children with severe malaria over hospitalization. **a**, **b**, **c** Representative plots from children receiving study gas with no interruptions to study gas. **d**, **e**, **f** Graphs showing MHb kinetics in children with a temporary interruption to study gas administration. **g**, **h**, **i** Graphs from children who had study gas permanently discontinued because they met criteria for acute kidney injury. **j**, **k**, **l**, Graphs from non-survivors

As this is the largest trial reported to date to administer nitric oxide at 80 ppm (the highest FDA approved dose), and methemoglobinemia is a known complication of iNO, we investigated the impact of 80 ppm iNO on MHb levels. This represents a secondary analysis of the study. The primary efficacy data are presented elsewhere [24]. MHb levels were assessed at scheduled times on a four hourly basis following study gas initiation. The mean time between initiation of study gas and the first gas check was 2.2 h, during which time there was a 2.3 fold-mean increase in MHb percent among children receiving iNO (mean MHb_Baseline_ = 1.8 to 4.1 %) but no change in children receiving placebo (mean MHb_Baseline_ = 1.7 to 1.8 %). Although there was considerable variability in MHb responses over time, MHb levels typically peaked and plateaued within 12–24 h of receiving nitric oxide (Fig. 1a). To illustrate the variability in MHb levels over time, representative plots of MHb levels over hospitalization were generated for a randomly selected subset (10 %) of study participants (*n* = 17; *n* = 7 placebo arm, *n* = 10 nitric oxide arm) (Fig. 1b). Representative graphs are shown for children who received study gas without interruption (A–C), children with temporary interruptions to gas flow (D–F), children who had study gas permanently discontinued because of acute kidney injury (G–I), and deaths (J–L) (Fig. 2).

#### MHb levels in fatal malaria

In the iNO RCT, we did not observe a significant difference between MHb levels assessed following randomization and mortality (MHb levels ≤2 % at screening was an eligibility requirement), *p* = 0.071 by Mann-Whitney U test. The majority of study deaths occurred in the first 48 h of hospitalization (*n* = 14 of 16 total deaths; [*n* = 8 in the placebo group, *n* = 6 in the iNO group]) with over half of study deaths (*n* = 8, 57.1 %) occurring before a second MHb measurement was taken. The mean time from study enrollment to death was 13 h. Because longitudinal data on MHb levels in non-survivors was limited, we were unable to explore differences in temporal trends in MHb levels between survivors and non-survivors.

Of children with multiple MHb measurements taken prior to death, there was one case of rising MHb prior to death. A 1 year old presented to the emergency department with a 3-day history of fever having received pre-referral treatment with chloramphenicol and intravenous quinine. The child was prostrate and comatose (Blantyre coma score = 2) with convulsions, prolonged capillary refill time (>3 s), jaundice, hypoglycemia, and severe anemia. At presentation, the patient had cough and age-related tachypnea (respiratory rate, 56/min) but no other signs of respiratory distress (nasal flaring, deep breathing, subcostal retractions). A diagnosis of severe malaria was made and the patient was treated with intravenous artesunate, dextrose, diazepam and phenobarbitone. Following enrollment in the clinical trial, the child was transferred to the study ward and study gas (room air) was initiated. On arrival to the study ward, the lactate level was 2.3 μmol/L and the MHb level was 0 %. Over the course of several hours, the child deteriorated clinically and developed respiratory distress with nasal flaring and intercostal and subcostal retractions and a progressive decline in Sp02 % to a nadir of 87 %. Supplemental oxygen was administered, but MHb levels continued to rise reaching 9.3 % before death (Fig. 2j). The cause of death was cardiopulmonary arrest.

Among children who died receiving iNO, only one had multiple MHb measurements taken prior to death (Fig. 2l).

## Discussion

In this study, we examined levels of MHb in two cohorts of children presenting to a regional pediatric referral hospital in Eastern Uganda. Although these studies represent distinct patient populations (there was no patient overlap between studies), the subjects were enrolled over the same 2 year period from the same catchment area. Overall, the children enrolled in the fever cohort were younger (according to the study design) and included all causes of fever, whereas all children in the iNO RCT had a diagnosis of severe malaria.

Among children enrolled in the fever cohort, there were no differences in MHb levels based on demographic characteristics (age, sex, or nutritional status). Infants less than 6 months represent a vulnerable group for methemoglobinemia for a number of reasons, including: a higher pH in the stomach that permits the growth of nitrate-reducing organisms (e.g. *Escherichia coli*, *Salmonella* spp.); immature NADH-methemoglobin reductase systems with reduced capacity to cope with oxidative stress (levels at birth are only 50–60 % of adult levels) [18, 25, 26]; the presence of fetal hemoglobin which is more readily oxidized to MHb than adult Hb [18]; and a higher consumption of water per unit body weight, which renders them susceptible to methemoglobinemia if exposed to nitrates through drinking water [27]. Despite these known risk factors in young children, we did not observe an age-dependent effect on MHb levels in our population of children hospitalized with fever.

Given the high rate of methemoglobinemia in our cohort (34 % of children in the fever cohort had MHb >2 %), Ugandan children may be at higher risk for methemoglobinemia than populations from high-resource settings as their environmental exposures may result in a higher set-point for MHb. A common cause of MHb in children is ingestion or contact with direct or indirect oxidizing agents (e.g. benzocaine, chloroquine, primaquine, sulfonamides, nitrites/nitrates, dapsone). As a result, we explored whether there was an association between known drug exposures (e.g. pretreatments with antibiotics and/or anti-malarials) and MHb levels. There was a non-significant increase in the odds of elevated methemoglobinemia in children that received pretreatment with any antibiotic (Table 2, *p* = 0.079). Due to the variability in antibiotic prescription and limitations in parental recall, we were limited in our ability to explore relationships between specific classes of drugs and MHb levels. Furthermore, we were unable to assess environmental exposure to other oxidizing agents in in this population (e.g., nitrates in water, or smoke inhalation through indoor biomass fuel use). However, a study evaluating nitrate levels in spring water from central Uganda reported 60 % (52/80) of water samples had nitrate levels exceeding the WHO maximum permissible levels [28]. In addition, widespread reliance on biomass fuel use (e.g., wood) in cooking stoves or open fires contributes to high levels of indoor air pollution [29]. It is estimated that 78 % of the Ugandan population resides in rural areas where 86 % use wood for cooking [29, 30]. Based on these findings, it is likely that environmental exposure to oxidants in our population exceeds WHO recommended levels.

We explored the association between clinical signs and symptoms at hospital presentation and MHb levels, and observed two general trends. First, children with impaired perfusion and acidosis (vomiting, delayed capillary refill, and elevated lactate) had significantly higher odds of methemoglobinemia after controlling for a set of potential confounders. These findings are consistent with previous studies in infants where methemoglobinemia was reported in the context of metabolic acidosis secondary to diarrhea and dehydration [31, 32]. In our cohort, we observed increased odds of methemoglobinemia associated with vomiting, but not diarrhea. Children with fever and diarrhea alone were excluded from the study. The prevalence of *P. falciparum* parasitemia in this cohort was high at 67 %. Therefore, the acidosis observed in this study may be attributable to malaria rather than dehydration and diarrhea, as metabolic acidosis is a common complication of malaria [33–35]. These data suggest that in conditions of increased inflammation, oxidative stress and acidosis, impaired reduction or reconversion of MHb to Hb may contribute to the elevated MHb levels observed in our cohort [18, 36, 37].

Methemoglobinemia was also seen in circumstances where red blood cells (RBCs) are affected: severe anemia, children with suspected or documented hemoglobinopathies (i.e. sickle cell disease or G6PD deficiency), and malaria. RBCs are particularly susceptible to oxidative damage as they carry oxygen in high concentrations and are continuously exposed to oxygen free radicals. As RBCs lack a nucleus, they are dependent on endogenous reduction systems that can degrade with repeated exposure to oxidants or RBC senescence [38]. Recent estimates of G6PD polymorphisms in Uganda show 20 % of the population carry the G6PD A-mutation [39], which are consistent with the range of estimates 15–32 % described elsewhere in Africa [40–42]. With increased oxidative stress on RBCs in G6PD deficiency, sickle cell disease, and other RBC polymorphisms, the capacity of endogenous reduction systems may be overwhelmed leading to increased MHb. In this population, rates of malaria were high with roughly two thirds of children admitted to hospital with fever having parasitologic evidence of malaria infection. Because quantitative estimates of malaria burden (either parasitemia or plasma HRP2 antigen levels) were not available in this cohort, it is difficult to estimate the fraction of fevers in children hospitalized attributable to malaria. Regardless, malaria was independently associated with increased odds of methemoglobinemia (OR (95 % CI), 1.34 (1.07, 1.69), *p* = 0.011), consistent with previous reports of methemoglobinemia in malaria [43–46]. Malaria is associated with increased oxidative stress from malaria-heme products and immune cell derived reactive oxygen species, both of which could promote oxidation of Hb to MHb [47, 48]. Finally, as children with severe anemia possess compromised oxygen carrying capacity, increased levels of MHb in the context of severe anemia may exacerbate reduced oxygen delivery resulting in metabolic acidosis and functional impairments in MHb reduction. Although elevated MHb was more common in non-survivors compared to survivors, it is likely that elevated MHb is a consequence of oxidative stress and acidosis in severe disease rather than mediating severe disease. However, in children with potentially symptomatic levels of MHb (e.g. the 3 % of children with MHb >10 % at admission in the fever cohort), MHb could exacerbate underlying disease processes and treatment may be warranted.

In the context of the clinical trial, 11 % of children assessed for eligibility were excluded for methemoglobinemia, which is considerably less than the fever cohort (34 %). The lower prevalence of methemoglobinemia in the iNO RCT may be due to a number of factors, including exclusion of children with known chronic illness (i.e., hemoglobinopathy). Furthermore, children otherwise eligible for the clinical trial were only excluded for methemoglobinemia if their MHb levels remained ≥2 % following stabilization (which included administration of fluids, transfusion in cases of severe anemia, and dextrose to treat/prevent hypoglycemia). As catabolism of sugars through glycolysis is a major source of substrate for the NADH-cytochrome-*b*_5_ reductase system, glucose levels must be in adequate supply for endogenous MHb reducing systems to respond [18]. Therefore, these stabilization measures may have contributed to a reduction in MHb levels in children allowing them to meet the eligibility criteria for the trial.

**Fig. 3:**
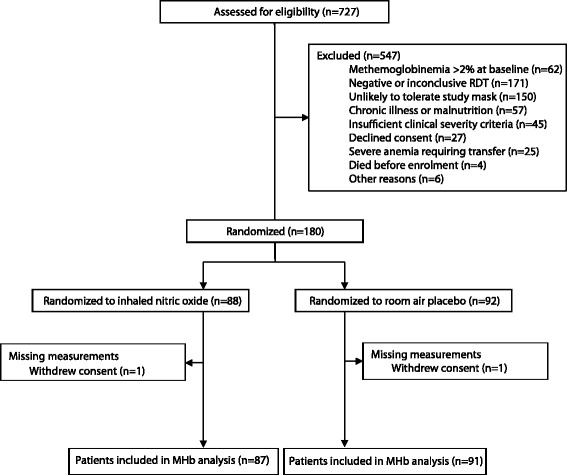
Flow chart of study enrolment for the randomized controlled trial

We were unable to evaluate the dose-dependent effect of iNO on MHb levels as all children randomized to receive iNO were started at 80 ppm. However, this is the largest trial to date to administer iNO at the maximum approved dose and we were able to evaluate the variability in MHb responses within subjects and the frequency of methemoglobinemia prompting study gas discontinuation. Despite the high doses of iNO administered, study gas was temporarily discontinued only five times for MHb >10 % (all children in the iNO group). We were able to re-start study gas for all children that had a MHb measurement that exceeded 10 % once the MHb returned to <7 % without having the MHb exceed 10 % again. It was not necessary to wean children off iNO, in contrast to studies administering iNO to neonates with hypoxic respiratory failure, as we did not observe any rebound effects (e.g. worsening oxygenation) following discontinuation of study gas [23, 49]. Overall, four hourly MHb checks were sufficient for monitoring iNO administration, with more frequent checks implemented in children when MHb levels approached 7 % so appropriate measures could be taken if levels exceeded 7 or 10 % (e.g. titrate or temporarily discontinue study gas). As seen in Fig. 3b, MHb levels fluctuated considerably within subjects over hospitalization in both trial arms. It is not clear whether these fluctuations were due to natural variations/regulatory responses in the endogenous reduction systems or were related to MHb measurement using the pulse co-oximeter. However, performance of non-invasive pulse co-oximetry has been previously compared to whole blood co-oximetry in children with sickle cell disease and showed acceptable clinical accuracy (bias of −0.22 % for MHb) [50]. The variations in MHb levels over hospitalization highlight the importance of frequent MHb monitoring during administration of nitric oxide.

When looking at illustrative graphs of MHb kinetics in children with and without study gas interruptions, the variability in responses is apparent (Fig. 1). We specifically included a panel of children who had gas permanently discontinued for acute kidney injury, as administration of iNO has been associated with a statistically elevated risk of developing renal dysfunction in critically ill adults [51]; however, the same association has not been observed in pediatric populations. In a recent retrospective analysis of acute kidney injury in this cohort, we found iNO was associated with an increased risk of acute kidney injury compared to placebo with a relative risk of 1.36 (95 % CI, 1.03-1.80), *p*=0.026. We did not observe differences in MHb levels in children who had gas discontinued for acute kidney injury compared to other children. Apart from the one child in the placebo arm of the trial who had increasing MHb levels prior to death, we did not observe elevated MHb among trial participants who died. However, the majority of participants died early in illness and there were limited kinetic data available. All children except one (Fig. 2l) died in the iNO arm died before repeated MHb measurements were taken.

After a decade of use in clinical practice, iNO has a well-established safety profile. In this study, we administered iNO in a low-resource setting in a non-intensive care setting with limited laboratory support. Using a commercially available handheld pulse co-oximeter, we determined the range of MHb levels for children admitted to hospital with fever in this population. Although MHb levels >2 % were observed in 34 % of children admitted with fever, methemoglobinemia was grounds for study exclusion in the iNO RCT for only 11 % of children following stabilization and repeat MHb assessment. In addition, we administered iNO at the highest approved dose in 87 patients with severe malaria and had to temporarily discontinue study gas for only a fraction of children (5.7 %) with MHb >10 %. Overall, the rates of study gas withdrawal were not different between the placebo and trial arm.

Our study strengths include two integrated studies encompassing a single catchment area: a large prospective observational study to describe MHb levels in children hospitalized with fever, and an intervention where a potent Hb oxidant was administered and MHb levels assessed. The clinical trial was randomized and double blinded using separate teams to monitor clinical care and study gas administration to ensure clinical decisions to withdraw gas were not affected by intervention. The study gas team used pre-set standard operating procedures to guide decisions regarding titration or withdrawal of study gas. Although we were limited in our ability to report dose-dependent effects of iNO on MHb levels, our study is the largest to administer iNO at the maximum approved dose and serves as an important addition to existing literature.

## Conclusions

Methemoglobinemia was a common complication among febrile Ugandan children admitted to hospital, and was associated with vomiting, metabolic acidosis, anemia, red blood cell polymorphisms, and malaria. Among children with severe malaria challenged with high-dose iNO, there was an increase in MHb levels, but rates of gas withdrawal for elevated MHb levels were low. These data suggest that iNO therapy, if clinically warranted, can be administered in low-resource settings provided appropriate monitoring is implemented.

### Key messages

- Methemoglobinemia (MHB >2 %) was a common feature among febrile Ugandan children admitted to hospital
- Elevated MHb was independently associated with vomiting, prolonged capillary refill time, and metabolic acidosis
- Children with anemia, red blood cell polymorphisms (e.g. sickle cell disease, G6PD deficiency), or malaria had elevated MHb at admission
- Administration of high-dose (80 ppm) inhaled nitric oxide in children with severe malaria resulted in increased MHb levels that plateaued 12–24 h after study gas initiation
- Inhaled nitric oxide was safe and well-tolerated in critically ill Ugandan children with severe malaria

## Abbreviations

BCS: Blantyre Coma Score
CM: Cerebral malaria
G6PD: Glucose-6-phosphate dehydrogenase
Hb: Hemoglobin
HRP2: Histidine-rich protein 2
iNO: Inhaled nitric oxide
IQR: Interquartile range
MHb: Methemoglobin
NO: Nitric oxide
OR: Odds ratio
pLDH: *Plasmodium* Lactate Dehydrogenase
ppm: Parts per million
RBC: Red blood cell
RCT: Randomized controlled trial
RDT: Rapid diagnostic test
SD: Standard deviation
WHO: World Health Organization
